# Evaluation of cost-effectiveness from the funding body's point of view of ultrasound-guided central venous catheter insertion compared with the conventional technique

**DOI:** 10.5935/0103-507X.20160014

**Published:** 2016

**Authors:** Danilo Teixeira Noritomi, Rogério Zigaib, Otavio T. Ranzani, Vanessa Teich

**Affiliations:** 1 Grupo de Cuidados Críticos Amil, Intensive Care Unit, Hospital Paulistano - São Paulo (SP), Brazil.; 2 Intensive Care Unit, Clinical Emergency Department, Hospital das Clínicas, Faculdade de Medicina, Universidade de São Paulo - São Paulo (SP), Brazil.; 3 Department of Pulmonology, Hear Instituto do Coração, Hospital das Clínicas, Faculdade de Medicina, Universidade de São Paulo - São Paulo (SP), Brazil.; 4 Insper - Instituto de Ensino e Pesquisa - São Paulo (SP), Brazil.

**Keywords:** Central venous cateteres/ economics, Ultrasonography/economics, Diagnostic techniques and procedures, Costs and cost analysis, Helath care costs, Unified Health System/economics

## Abstract

**Objective:**

To evaluate the cost-effectiveness, from the funding body's point of view, of real-time ultrasound-guided central venous catheter insertion compared to the traditional method, which is based on the external anatomical landmark technique.

**Methods:**

A theoretical simulation based on international literature data was applied to the Brazilian context, i.e., the Unified Health System (*Sistema Único de Saúde* - SUS). A decision tree was constructed that showed the two central venous catheter insertion techniques: real-time ultrasonography versus external anatomical landmarks. The probabilities of failure and complications were extracted from a search on the PubMed and Embase databases, and values associated with the procedure and with complications were taken from market research and the Department of Information Technology of the Unified Health System (DATASUS). Each central venous catheter insertion alternative had a cost that could be calculated by following each of the possible paths on the decision tree. The incremental cost-effectiveness ratio was calculated by dividing the mean incremental cost of real-time ultrasound compared to the external anatomical landmark technique by the mean incremental benefit, in terms of avoided complications.

**Results:**

When considering the incorporation of real-time ultrasound and the concomitant lower cost due to the reduced number of complications, the decision tree revealed a final mean cost for the external anatomical landmark technique of 262.27 Brazilian reals (R$) and for real-time ultrasound of R$187.94. The final incremental cost of the real-time ultrasound-guided technique was -R$74.33 per central venous catheter. The incremental cost-effectiveness ratio was -R$2,494.34 due to the pneumothorax avoided.

**Conclusion:**

Real-time ultrasound-guided central venous catheter insertion was associated with decreased failure and complication rates and hypothetically reduced costs from the view of the funding body, which in this case was the SUS.

## Abstract (pt)

**Objetivo:**

Avaliar o custo-efetividade da inserção de cateter venoso central guiada por ultrassonografia em tempo real, em comparação com a técnica tradicional, que é baseada na técnica de reparos anatômicos externos, sob a perspectiva da fonte pagadora.

**Métodos:**

Uma simulação teórica, baseada em dados de literatura internacional foi aplicada ao contexto brasileiro, ou seja, ao Sistema Único de Saúde (SUS). Foi estruturada uma árvore de decisão, que apresentava as duas técnicas para inserção de cateter venoso central: ultrassonografia em tempo real *versus* reparos anatômicos externos. As probabilidades de falha e complicações foram extraídas de uma busca nas bases PubMed e Embase, e os valores associados ao procedimento e às complicações foram extraídos de pesquisa de mercado e do Departamento de Informática do Sistema Único de Saúde (DATASUS). Cada alternativa de passagem do cateter venoso central teve um custo calculado por meio do seguimento de cada um dos possíveis caminhos da árvore de decisão. A razão de custo-efetividade incremental foi calculada considerando-se a divisão do custo incremental médio da técnica de ultrassonografia em tempo real comparada à técnica de reparos anatômicos externos pelo benefício incremental médio, em termos de complicações evitadas.

**Resultados:**

O custo final médio avaliado pela árvore de decisão, considerando a incorporação da ultrassonografia em tempo real e a redução de custo por diminuição de complicações, para a técnica de reparos anatômicos externos foi de R$262,27 e, para ultrassonografia em tempo real, de R$187,94. O custo incremental final foi de -R$74,33 por cateter venoso central. A razão de custo-efetividade incremental foi -R$2.494,34 por pneumotórax evitado.

**Conclusão:**

A inserção de cateter venoso central com auxílio de ultrassonografia em tempo real esteve associada à diminuição da taxa de falhas e complicações, além de hipoteticamente reduzir custos na perspectiva da fonte pagadora, no caso o SUS.

## INTRODUCTION

The central venous catheter (CVC) is currently regarded as one of the fundamental tools in hospital medical practice. The indications for its use are numerous: administration of vasopressors, hemodynamic monitoring (measurement of central venous pressure and venous oxygen saturation) and when peripheral venipuncture in not possible. Currently, more than 5 million CVC are used in the United States per year.^(1)^ The Department of Information Technology of the Unified Health System (Departamento de Informática do Sistema Único de Saúde - DATASUS), maintains a national database that contains procedures reimbursed by the SUS, which shows that 103,922 CVC were used in Brazil in 2013.^(2)^ This number may be underestimated, as the database does not account for procedures reimbursed by the supplementary health system.

The use of CVC is not free of complications, either in terms of insertion or maintenance of the device.^(3)^ Traditionally, the devices are inserted using the external anatomical landmark technique (EALT), in which observation and palpation of anatomical landmarks serve as a reference for deciding the best place to make the puncture. However, this technique is subject to error, mainly because of anatomical variations in the population.

Recently, the use of real-time ultrasound-guided (RTUSG) CVC has been incorporated into medical practice^(4)^ This method has become popular over the last decade, and a series of studies have demonstrated its safety and applicability as well as a reduction in complications of CVC insertion.^(4,5)^

However, the fact that the incorporation of technologies can result in significantly increased costs in health care without there necessarily being a proportional improvement in the quality of care offered to the public must be considered. In part, this discrepancy may be due to the incorporation of technologies that are ineffective or too costly. Despite the scientific sustainability of ultrasound-guided CVC insertion due to its being an effective procedure in reducing complications, systemic incorporation of this technology presents a challenge. Incorporating a new technology that requires significant resources can result in a lack of resources for other care activities that are already in place. In practice, the health manager finds little evidence to support his decision within the scientific literature and is often guided by non-measurable elements, which leads to the possibility of cognitive bias.^(6,7)^ A recent survey showed that the incorporation of health technology in hospitals is rarely based on any cost-effectiveness analysis.^(8)^

The objective of this study was to evaluate from the perspective of the funding body, in this case the SUS, the cost-effectiveness of incorporating a relatively new clinical practice - RTUSG central venous catheter insertion - compared with the traditional method based on EALT.

## METHODS

**Figure 1:**
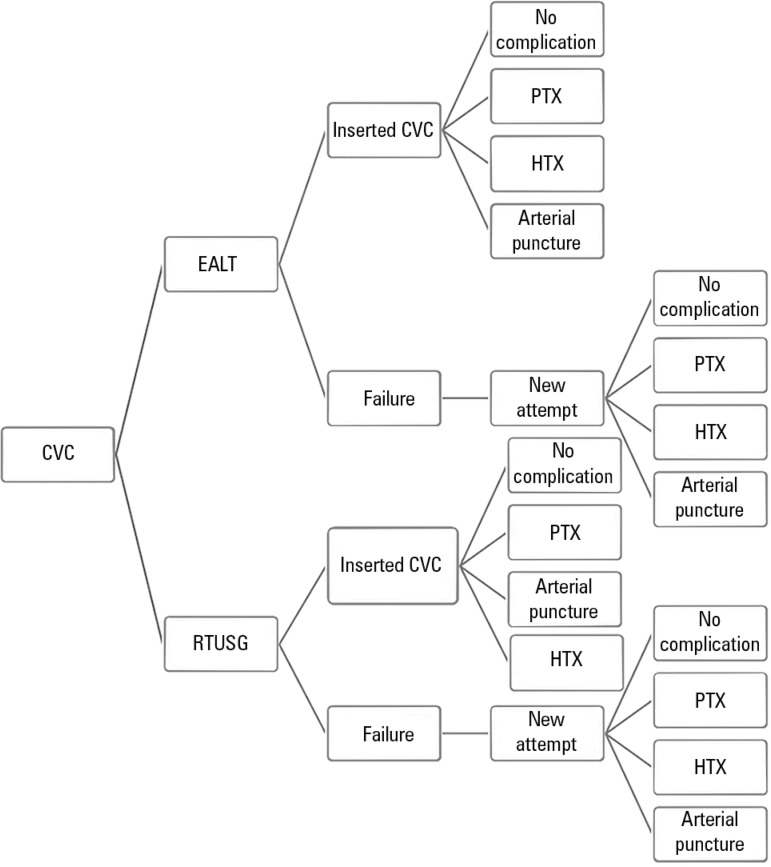
Decision tree. CVC - central venous catheter; EALT - external anatomical landmark technique; RTUSG - real-time ultrasound-guided technique; PTX - pneumothorax; HTX - hemothorax.

This study consisted of a theoretical simulation based on data from the international literature applied to the Brazilian context. A decision tree was constructed that presented both alternatives for CVC insertion and that then followed the possible outcomes that could be observed in patients. The proposed model is shown in figure 1.

The model starts with the possibility of CVC insertion using one of two techniques: EALT or RTUSG. Both techniques can involve CVC insertion failure or success. Insertion can be free of complications or not. The three most frequent complications are pneumothorax, hemothorax and arterial puncture. In our analysis, we chose not to consider the outcome death because this is not an expected complication of CVC insertion and because costs associated with this complication have not been described. In case of failure, a new attempt was made, and the possibilities were repeated. What distinguished EALT from RTUSG was the probabilities assigned to each outcome, with the most probable unfavorable outcomes applying to the EALT branch.^(4)^

### Likelihood of complications

To construct the described theoretical model, it was necessary to map the probability of each outcome/complication after attempting to insert the CVC with the use of each technique. To that end, a review was conducted of the scientific literature to find the best estimates of the efficacy of the methods that could be used as a basis for the cost-effectiveness simulation in a national context. We conducted a search of the PubMed and Embase databases, from the first entries in these databases up to August 2013, using the Boolean search method, with the following terms: ("central venous line" OR "central venous line insertion" OR "central venous catheter" OR "central venous access" OR "central line insertion" OR "CVC" OR "IJV" OR "FV") AND ("ultrasonography" OR "echography" OR "ultrasound" OR "ultrasonic" OR "image guidance" OR "image guided") AND ("mechanical complication" OR "pneumothorax" OR "cost-effectiveness" OR "cost" OR "length of stay" OR "los" OR "arterial puncture" OR "hematoma" OR "haematoma" OR "hemothorax" OR "haemothorax"). Among the studies found, a systematic review and meta-analysis by Wu et al.^(4)^ met the inclusion criteria for our research, as it was the most recent, had a rigorous methodology and provided the parameters required for building our comparative analysis of the two methods.^(9-31)^ We therefore chose to use the results of that study as a basis for our analysis.

### Costs

The following describes the methods used to calculate the costs of the resources associated with CVC insertion and of the treatment of complications. To define the costs associated with CVC insertion and its complications at a national level, we interviewed a convenience sample of five qualified experts in intensive care medicine who had extensive experience in CVC insertion and who worked mostly in Brazilian public hospitals.

- CVC insertion: the cost of CVC insertion was estimated using the corresponding DATASUS codes,^(3)^ considering the weighted mean reimbursement amount when inserting double-lumen and single-lumen CVCs and short-term hemodialysis catheters in the proportions 80%, 10% and 10%, respectively, according to the expert's opinion.
- Cost of the ultrasound device: The cost associated with ultrasound is incurred at the time the equipment is purchased by the hospital. The cost associated with each procedure that uses an ultrasound device was calculated by projecting the expected number of cases of CVC insertion in a hospital. The cost per procedure associated with the ultrasound device is then the result of dividing the cost of the equipment by the total number of tests that this device performs before obsolescence (obsolescence is considered to be after 5 years). For the purpose of this analysis, we considered a hypothetical service with insertion of 325 CVCs per year, keeping in mind that this number can vary greatly among services. The cost was estimated in August 2013 according to market research and consulting public tenders for purchase of the device.
- Cost of protective devices: obtained from market research of device suppliers.
- Cost of complications: the cost of interventions needed to treat complications. Each complication requires a potential set of interventions. The amount of each resource used for the treatment of complications (e.g., probability of thoracotomy for the treatment of pneumothorax) was estimated by consultation with experts. Then, to calculate the cost of a particular type of complication (e.g., pneumothorax), the probability that the patient would require each potential intervention was calculated, and the result was multiplied by the cost of the intervention. Specifically, to calculate the cost of blood products used in hemothorax treatment, the various costs involved were considered, such as blood collection, transfusion tests and transfusion itself - from blood donation to processing and administration of the blood product. The cost of a particular complication type is the sum of each of these values associated with each therapeutic intervention.

### Comparison of methods

The total cost associated with each alternative CVC insertion method was calculated by adding the costs of following each of the possible decision tree paths, weighted by the probability of its occurrence. The incremental cost-effectiveness ratio (ICER) was calculated by dividing the mean incremental cost of the RTUSG technique compared to the EALT technique by the mean incremental benefit, in terms of avoided complications, according to equation 1.

$\mathrm{ICER}=\frac{\mathrm{△C}}{\mathrm{△E}}\;\;\;\;\;\;\;\;\;\;\;\;\;\;\;\;\;\;\;\;\;\;\;\mathrm{Equation}1$△C - incremental cost of the RTUSG technique compared to the
EALT technique; △E= incremental effectiveness of the RTUSG technique
compared to the EALT technique, in terms of avoided
complications.

To compare methods, a hypothetical base case was analyzed, namely, insertion of 325 CVC per year over a period of 5 years in a service that performs CVC insertion using the EALT technique and another with the same characteristics carrying out the insertions using the RTUSG technique.

### Sensitivity analysis

Key parameters were varied in the univariate sensitivity analysis to evaluate the uncertainty effect of these parameters on the results of the analysis. The main parameters varied were the pneumothorax rate associated with the standard technique (0.5 to 2 times the central estimate) and the mean number of CVC inserted per service (0.25 to 4 times the central estimate). Pneumothorax (ICER per avoided pneumothorax) was chosen as the primary outcome in the sensitivity analyses because it is the most common complication with an associated cost.

## RESULTS

### Efficacy

Table 1 was constructed based on the selected meta-analysis, which confirmed better efficacy of CVC insertion with RTUSG.

**Table 1:** Probability of outcomes and incremental effectiveness of the two methods

**Outcome**	**EALT (%)**	**RTUSG (%)**	**Incremental effectiveness %**
Failure rate	10.80	1.30	9.50
Pneumothorax rate	3.09	0.11	2.98
Hemothorax rate	2.60	0.00	2.60
Arterial puncture rate	10.80	1.50	9.30
No complications	83.51	98.39	14.88

EALT - external anatomical landmark technique; RTUSG - real-time ultrasound-guided technique.

### Costs

The estimated CVC insertion procedure cost considered three possible different types of catheter insertion in the ICU, as shown in table 2. The mean cost per insertion procedure using the EALT technique was R$95.64.

**Table 2:** Weighted cost of central venous catheter insertion

**Resource**	**Patients (%)**	**Unit value (R$)**	**Weighted value (R$)**
Single lumen	10	112.48	11.25
Double lumen	80	85.00	68.00
Dialysis	10	163.89	16.39
Weighted mean			95.64

According to market research, the cost of an ultrasound machine is R$45,000.00. The effect of the device value on each procedure was R$87.69. The cost of protective devices was estimated at R$60.00 per CVC inserted. Therefore, the additional cost of CVC using the RTUSG method would be R$147.69; this is not taking into consideration that ultrasound reduces complications.

The cost of complications was calculated based on the expert panel opinion and on data from the funding body, SUS. Detailed costs are as shown in tables 3 and 4.

**Table 3:** Mean estimated cost of treatment of a pneumothorax case

**Resources**	**Patients (%)**	**Quantity**	**Unit value (R$)**	**Total cost (R$)**
Physiotherapy	100	15	4.67	70.05
Thoracotomy with closed pleural drainage	75	1	2,512.46	1,884.35
Thoracentesis/pleural drainage	5	1	54.97	2.75
Exploratory thoracotomy	1	1	3,553.82	35.54
Pneumorrhagia	1	1	3,130.14	21.91
Videothoracoscopy	1	1	1,773.61	12.42
Total				2,027.01

**Table 4:** Mean estimated cost of treatment of a hemothorax case

**Resources**	**Patients (%)**	**Quantity**	**Unit value (R$)**	**Total cost (R$)**
Physiotherapy	100	15	4.67	70.05
Thoracotomy with closed pleural drainage	90	1	2,512.46	2,261.21
Red cell concentrate transfusion	40	2	91.11	72.89
Thoracentesis/pleural drainage	30	1	54.97	16.49
Exploratory thoracotomy	15	1	3,553.82	533.07
Videothoracoscopy	30	1	1,773.61	532.08
Plasma transfusion	20	2	91.11	36.44
Intrathoracic retained clot treatment	10	1	2,582.02	25.82
Platelet transfusion	10	6	91.11	54.67
Total				3,602.73

We considered the treatment cost of a hematoma due to puncture to be negligible.

### Cost-effectiveness analysis

Using the decision tree and considering the incorporation of the new technology and the concomitant cost reduction by reducing complications, the final mean estimated costs were R$262.27 for the EALT technique and R$187.94 for the RTUSG. The final incremental cost was therefore -R$74.33. Table 5 shows the cost-effectiveness results based on the additional cost of avoided complications when using the RTUSG technique.

**Table 5:** Cost-effectiveness results

**Incremental cost-effectiveness ratio**	**Results (R$)**
Incremental cost per case of pneumothorax avoided	-2,494.34
Incremental cost per case of hemothorax avoided	-2,858.90
Incremental cost per case of hematoma avoided	-799.26

ICER - incremental cost-effectiveness ratio.

### Base case analysis

**Figure 2:**
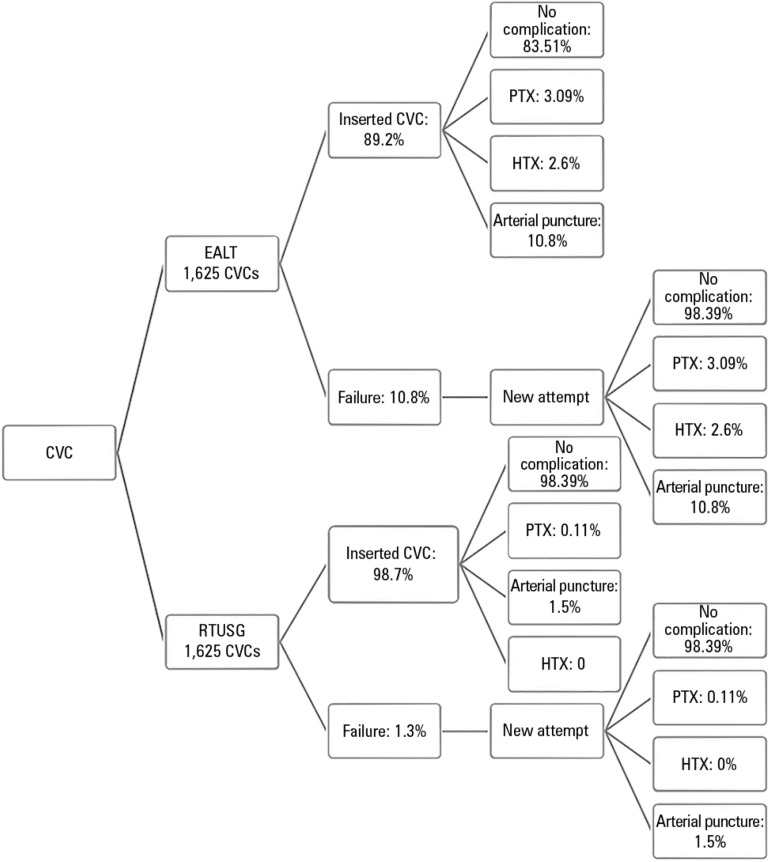
Flow chart with percentages attributed to complications in each CVC insertion technique. CVC - central venous catheter; EALT - external anatomical landmark technique; RTUSG - real-time ultrasound-guided technique; PTX - pneumothorax; HTX - hemothorax.

We considered a 5-year monitoring period for two services performing 325 CVC insertions per year, totaling 1,625 CVC for each service over 5 years. The center using the EALT technique had CVC and complication costs; the center using the RTUSG technique, in addition to these costs, also had the cost of the ultrasound device and disposable materials used to perform the technique. Additionally, all RTUSG centers must acquire a new device every 5 years. The increased effectiveness and reduced complications of CVC insertion afforded by the RTUSG technique led to a reduction in costs in the order of R$100,000.00. The results are represented in figure 2 and table 6.

**Table 6:** Number of complications and costs associated with each central venous catheter insertion technique

**Description**	**Number of events and/or costs**
EALT (CVC: 325/year - 1,625/5 year)	
Events (in 5 years)	
Failure (more than 1 insertion)	176
Pneumothorax	50
Hemothorax	43
Arterial puncture	176
Total cost (in 5 years)	
Catheters (R$)	170,508.43
Complications (R$)	253,996.58
Disposable materials	0
Ultrasound device	0
Total (R$)	424,505.01
RTUSG (CVC: 325/year - 1625/5 years)	
Events (in 5 years)	
Failure (more than 1 insertion)	21
Pneumothorax	2
Hemothorax	0
Arterial puncture	24
Total cost (in 5 years)	
Catheters (R$)	157,435.40
Complications (R$)	3,623.28
Disposable materials (R$)	98,767.50
Ultrasound device (R$)	45,000.00
Total (R$)	324,816.18

### Sensitivity analysis

Varying the rate of pneumothorax encountered using the anatomical method from 1.5% to 4.5% produced a variation in the ICER of -R$2,977.50 to -$2,256.44. In turn, varying the mean number of CVC insertion procedures performed each year in a given service from 81 to 1,300 per year produced a variation in the ICER of between R$264.06 and -R$3,266.14. This sensitivity analysis suggests a point of equilibrium of approximately 87 insertions per year. This was the minimum number of insertions with a single device after which CVC insertion using ultrasound would not incur an extra cost to the health system.

## DISCUSSION

According to our model, RTUSG CVC insertion is a cost-saving intervention and prevents complications, as shown by the negative ICER in the prevention of complications.

Cost-effective interventions are considered those with increased care costs below a threshold arbitrarily defined as acceptable. When the intervention is able to reduce mortality, there are some suggested thresholds. The World Health Organization (WHO) recommends that an intervention is highly cost-effective if the incremental cost per additional year of life adjusted for quality of life does not exceed the per capita gross domestic product (GDP) of the country in question. An intervention is cost-effective if the ICER is one to three times the per capita GDP; if it exceeds three times the per capita GDP, it is not a cost-effective intervention.^(32)^ This is a concrete element upon which to guide the administrator's decision, given that the Brazilian GDP per capita in 2013 was approximately US$11,700.00 (close to R$25,000.00).^(33)^ However, studies comparing the RTUSG technique to the EALT technique do not consider the possibility of a change in mortality with the acquisition of the new technology.

We can state with some certainty that the new technology is cost-saving for a number of reasons. In terms of cost increase, the major determinants of cost tend to become progressively less than those estimated in the base case, as there is a downward trend in device and disposable material costs over the years, given the normal technological evolution in this area. Furthermore, we did not consider the possibility of sharing equipment, which minimizes the cost of the intervention, rendering it even more cost-saving. Still on that side of the equation, the device usage rate in the baseline scenario was quite low, and the obsolescence interval was relatively short. Regarding the costs saved related to complications, values were determined primarily by inarguably necessary components in most cases (such as thoracostomy with pleural drainage).

In our sensitivity analysis, if the number of CVC inserted by RTUSG was below 87 per year, the number of complications would still be much smaller, but costs would not be saved. This situation is usually found when incorporating new health technologies. However, if the annual number of CVC insertions per center were lower, it would be expected that the chance of complications associated with CVC insertion would be higher due to the lower volume of insertion and training. Therefore, although we did not simulate this scenario (for example, fewer CVC inserted annually, by center, leading to an increased risk of complications), we can speculate that the use of RTUSG would be effective and cost-saving even in scenarios with low use. Moreover, the sensitivity analysis related to pneumothorax complications always showed a negative ICER.

Our model offers important information about the decision to incorporate the technology in question in the SUS, but, of course, it does not end the discussion. From a strict cost-effectiveness point of view, the decision to incorporate the technology is clearly favorable, given that the intervention was not only cost-effective but also cost-saving (the base case analysis showed a reduction of R$100,000.00 in resources used over 5 years). A simultaneous reduction in complications and costs would therefore be observed. However, we should also consider the weaknesses of the analysis, some of which have already been highlighted, and factors that were not taken into account. One of these factors is the immediate impact on the budget. Although the intervention may save resources over time, the funding body must assume a cost that takes place in the present, and the manager should consider whether he is able to pay such an amount immediately. Another point to consider is the usefulness of an ultrasound machine in the intensive care setting for other interventions, such as hemodynamic and cardiovascular evaluations and procedures such as paracentesis, pericardiocentesis and chest puncture. The use of the device can improve and add safety to patient care;^(4,5)^ this is another element not measured in the study that the manager needs to consider.

This study has some limitations. The occurrence rates of events occurring with the EALT and RTUSG techniques were drawn from an international meta-analysis that included no experience from our environment. However, because CVC insertion is a standard technique across the world, we do not believe that Brazilian rates would be much different from those observed in the meta-analysis. In addition, the meta-analysis included only randomized studies, which may underestimate complication rates because such studies usually have more controlled samples than a hospital or clinic will encounter. However, these randomized studies included physicians with and without experience, which may have diluted any effect caused by the better results observed in randomized studies. The cost estimate associated with each complication involved some assumptions, such as the likelihood of the need for each therapeutic intervention. These probabilities were estimated using the experience of an expert panel and are subject to error. However, we consider the values to be relatively conservative within the consensus range. Furthermore, all directly calculated costs (cost of materials, equipment, etc.) can vary greatly over time and affect the analysis presented at this time. Finally, the economic analysis was performed with data extracted from DATASUS and is based on the SUS's perspective. It should therefore be interpreted in this context, without the possibility of direct extrapolation of these results to other settings.

## CONCLUSION

Real-time ultrasound-guided central venous catheter insertion was associated with decreased failure and complication rates and hypothetically reduced costs from the point of view of the funding body, which in this case was the Brazilian SUS.
